# The relative importance of metabolic rate and body size to space use behavior in aquatic invertebrates

**DOI:** 10.1002/ece3.11253

**Published:** 2024-05-20

**Authors:** Milad Shokri, Vanessa Marrocco, Francesco Cozzoli, Fabio Vignes, Alberto Basset

**Affiliations:** ^1^ Laboratory of Ecology, Department of Biological and Environmental Sciences and Technologies University of Salento Lecce Italy; ^2^ National Biodiversity Future Center (NBFC) Palermo Italy; ^3^ LifeWatch ERIC, Service Centre, Campus Ecotekne Lecce Italy; ^4^ Research Institute on Terrestrial Ecosystems (IRET) – National Research Council of Italy (CNR) via Salaria Monterotondo Scalo (Rome) Italy

**Keywords:** body size, energy requirements, foraging behavior, metabolic rate, resource use, space use

## Abstract

Elucidating the underlying mechanisms behind variations of animal space and resource use is crucial to pinpoint relevant ecological phenomena. Organism's traits related to its energy requirements might be central in explaining behavioral variation, as the ultimate goal of a forager is to fulfill its energy requirements. However, it has remained poorly understood how energy requirements and behavioral patterns are functionally connected. Here we aimed to assess how body mass and standard metabolic rate (SMR) influence behavioral patterns in terms of cumulative space use and time spent in an experimental patchy environment, both within species and among individuals irrespective of species identity. We measured the behavioral patterns and SMR of two invertebrate species, that is, amphipod *Gammarus insensibilis*, and isopod *Lekanesphaera monodi*, individually across a range of body masses. We found that species of *G. insensibilis* have higher SMR level, in addition to cumulatively exploring a larger space than *L. monodi.* Cumulative space use scaled allometrically with body mass, and it scaled isometrically with SMR in both species. While time spent similarly in both species was characterized by negative body mass and SMR dependence, it was observed that *L. monodi* individuals tended to stay longer in resource patches compared to *G. insensibilis* individuals. Our results further showed that within species, body mass and metabolic rate explained a similar amount of variation in behavior modes. However, among individuals, regardless of species identity, SMR had stronger predictive power for behavioral modes compared to body mass. This suggests that SMR might offer a more generalized and holistic description of behavioral patterns that extend beyond species identity. Our study on the metabolic and body mass scaling of space and resource use behavior sheds light on higher‐order ecological processes such as species' competitive coexistence along the spatial and trophic dimensions.

## Graphical abstract

Our study explores how metabolic rate and body size are related to space use behavior, using invertebrate as model species. It shows that metabolic rate, encompassing variations related to species‐specific characteristics, is associated with behavior beyond mere size.

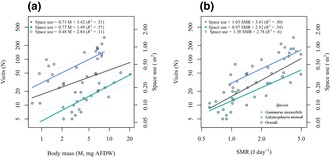

## 1 INTRODUCTION

Space and resource use behavior differ considerably within and among species which has significant implications in ecological processes, for example, competitive coexistence and consumer‐resource community (Auer et al., 2020; Shokri et al., 2024). However, despite its importance in ecology and seminal studies drawing attention to this field (e.g., Basset, 1995; Charnov, 1976; MacArthur & Pianka, 1966; Stephens & Krebs, 1986), the mechanism underpinning the expression of behavioral variability remains the subject of active research debate (Biro & Stamps, 2010; Careau & Garland, 2012). Animals use space for a variety of purposes, for example, sheltering from predators and finding mates (Jermacz et al., 2022; Zhang et al., 2021). However, at the fundamental level, space and resource use behavior is aimed at fulfilling organisms' energy requirements (MacArthur & Pianka, 1966; Stephens & Krebs, 1986), making it intuitive that an energetic perspective could provide a mechanistic view of behavioral patterns.

Body mass, which is intimately associated with and one of the main components of an organism's energetic requirements, has received considerable attention as a proxy for these requirements (Brown et al., 2004; Peters, 1983). Animal body mass has often been employed to predict various types of behavior, including patch giving up and space use behavior (e.g., Basset et al., 2012; Brose, 2010; Cozzoli et al., 2022; McNab, 1963). This is mainly because motile animals need to adjust their space and resource use behavior to meet their mass‐dependent needs (Brown et al., 2004). For instance, studies have shown that patch abandoning decisions are correlated with animal body mass, with larger individuals leaving resource patches earlier than smaller ones (Cozzoli et al., 2018; Kotler & Brown, 1990). It follows that at the increase of the individuals' body mass, animals generally need larger home ranges and exploit a resource patch only if and for as long as it allows high ingestion rates (Basset, 1995; McNab, 1963). Furthermore, larger animals generally have higher dispersal ability (Cloyed et al., 2021; Hirt et al., 2017) and lower locomotion costs per unit of body mass, which contribute to their greater propensity to explore the surrounding environment (Dial et al., 2008).

Despite the common use of body mass as a predictor of behavior, empirical studies have shown significant variations in energy metabolism within and among species that are similar in body mass (Burton et al., 2011; Shokri et al., 2019). Such differences may arise from the joint effect of several factors such as taxonomic affiliations, life history, niche breadth, body morphology, and mobility, which might hinder the predictive power of body mass in predicting the behavioral patterns (Killen et al., 2010, 2016). For instance, among species with similar body mass, swimmers, or more active ones, require more energy to maintain high athletic performance than sedentary species (Killen et al., 2016).

In turn, the metabolic rate as the measure of energy requirements, by setting a common currency of energy, can improve generalization in traits related to energy acquisition such as foraging decision and space use behavior (Shokri et al., 2024). Importantly, metabolic rate integrates a variety of typical trait proxies for energy acquisition and allocation in animals, for example, body mass, diet, mobility, and life history to yield a fine scale suite of continuous quantities (Brandl et al., 2022; Glazier, 2015). Growing evidence indicates that animals within and between species differ in their rate/capacity to generate and sustain the minimum level of energy requirements, that is, referred to as standard metabolic rates (SMR) in ectotherms (Auer et al., 2018), and that this difference may in turn be a predictor of the behavior patterns (Biro & Stamps, 2010; Careau et al., 2008; Metcalfe et al., 1995). In other words, differences in maintenance metabolic rates among animals might dictate the optimal expression of behaviors associated with energy intake and expense (Mathot et al., 2015). This follows a positive feedback loop between metabolic rate and behavior; as such, having a higher metabolic rate would require animals to intake more resources to sustain and fuel their larger metabolic requirements (sensu Biro & Stamps, 2010). It follows that higher metabolic rates would necessitate a larger area to explore for resource gathering. However, it has also been hypothesized that a higher metabolic rate can come with a higher maintenance cost and less energy available to spend on active behavior (sensu Careau et al., 2008). Therefore, while metabolic rate is expected to have a significant influence on behavioral patterns, a full consensus has not yet been emerged regarding the nature of the functional relationship between them (Careau & Garland, 2012).

Here, we aimed to investigate how behavioral modes, in terms of space and time use, are connected to body mass and metabolic rate in a resource‐patchy environment within and among two ecologically similar invertebrate species (sensu Harper et al., 1961) with differing natural histories. We further investigated the predictive power of body mass and metabolic rate on variations in behavioral modes, both within species as well as among individuals irrespective of species identity. This approach could help to determine which of these traits provides a more generalized and holistic explanation for behavioral patterns. The experiment was carried out on aquatic invertebrate species, amphipod *Gammarus insensibilis*, and isopod *Lekanesphaera monodi* across a wide range of body masses. Concerning macroinvertebrates, currently little is known about their space and resource use behavior (but see Jermacz et al., 2015, 2020, Mancinelli, 2010). Using the full body mass range of the two species and tracking their individual behavioral patterns and SMR allowed us to disentangle the links of SMR and body mass (M) to the descriptors of time and space use behavior.

## 2 MATERIALS AND METHODS

### 2.1 Model species

For this study, we used two common aquatic invertebrates: *Gammarus insensibilis* (Stock, 1966) and *Lekanesphaera monodi* (Arcangeli, 1934). The selection of these model species was based on their year‐round presence, feed on similar resources, often coexist, having similar size ranges, and the ease of their manipulation and handling in laboratory experiments. However, although both species belong to Malacostraca, they differ in their taxonomic orders, which may also reflect differences in their life histories. *Gammarus insensibilis* is a widespread Atlantic‐Mediterranean amphipod species living in coastal and transitional waters (Costello et al., 2001). In the reproductive process of *Gammarus* sp., eggs are brooded within the marsupium of females. After hatching, juveniles undergo initial development and are released at approximately 1 mm in body length. They reach maturity at 0.4 cm (Longo & Mancinelli, 2014) and can grow to a maximum length of about 2 cm (Tillin & White, 2017). These amphipods have a maximum lifespan of up to 1 year, depending on environmental conditions (Janssen et al., 1979). Their body are laterally compressed and they are known as active swimmer (Ruppert et al., 2004). *Gammarus* sp. are widely distributed species due to their broad trophic repertoire, foraging flexibility, and migration ability, which allows them to invade and colonize ecosystems (Gerhardt et al., 2011; Shadrin et al., 2022). Species of *Gammarus* sp. feed preferentially on fungi with high protein content, colonized on decomposed leaf litter (Bärlocher & Kendrick, 1973). However, they exploit less palatable microorganisms or even the matrix of leaves when their preferred food is in short supply. *Gammarus* sp. can reach a daily consumption rate of about 46%–103% of their body size (Berezina, 2007), and a gut throughput time of 45–59 min at 14°C with unlimited resource availability (Welton et al., 1983).

*Lekanesphaera monodi* is an isopod species with a distribution range from the North Sea to the Mediterranean Seas. It inhabits both marine and transitional water ecosystems and often coexists with Gammarid species. In *Lekanesphaera* sp., eggs are brooded within the marsupium of the female and, after hatching, the juveniles are released. These isopods reach a body length of 0.3 cm at maturity (Longo & Mancinelli, 2014), have an average body length of 1.2 cm (Jacobs, 1987), and can have a lifespan of up to 1 year (Ellis, 1961). They are known to be relatively sedentary organisms, moving by crawling on the substrate, swimming short distances, and characterized by their dorso‐ventrally compressed bodies (Longo et al., 2016; Mancinelli, 2010). The *Lekanesphaera* sp. species, like *Gammarus* sp., feed on fungi found in leaf litter. They can achieve a daily consumption rate of up to 40%–80% of their body mass (Rossi, 1985; Smock & Harlowe, 1983).

Both model species inhabit transitional water ecosystems, which are naturally subject to wide temperature variations. The upper thermal limit of *Gammarus* sp. has been recorded at 33°C (Verberk et al., 2018), whereas *Lekanesphaera* sp. has been documented to withstand temperatures up to 34°C (Castañeda & Drake, 2008).

### 2.2 Species collection and acclimation

Specimens of *G. insensibilis* and *L. monodi* were collected from closely situated transitional water bodies in the Southwest of the Adriatic Sea, in Italy; the Cesine natural reserve area (40.36° N, 18.33° E), and Acquatina (40.44° N, 18.23° E), respectively. The authorization for the collection of specimens was issued by the competent authority World Wildlife Fund for Nature (Italy) and the University of Salento. After collection, the specimens were transferred alive in the Biodiversity Ecosystem Functioning Laboratory (*BIO4IU*) at University of Salento by thermo‐insulated containers filled with water from the sampling sites and aerated during transport. The specimens of *G. insensibilis* and *L. monodi* were maintained in the laboratory aquaria, similar to what they were experiencing at the field sampling site and acclimated for 2 weeks at 18°C. The acclimation temperature was selected to correspond to the water temperature at the time of collection (i.e., 18 ± 0.5°C). This temperature also closely approximates the average annual water temperature in these water bodies. The specimens of both species were fed conditioned leaves of *Phragmites australis* (Cav.) Trin. ex Steud ad libitum during maintenance, reflecting their trophic resources in the natural environment (Basset et al., 2001).

Prior to the experiment, specimens were sorted by sex under a Nikon stereoscope (SMZ1270). Only males, corresponding to the adult stage, were selected for the metabolic and behavioral measurements. This was because oocyte production in females may induce higher beyond‐size variability in energy requirements and behavior, and focusing on adult stages helps minimize variation due to different ontogenic stages (Glazier et al., 2011; Normant et al., 2007).

### 2.3 Preparation of trophic resources

Leaves of *Phragmites australis* (Cav.) Trin. ex Steud were collected at the site of the specimens' collection, cut into approximately 10 cm lengths, dried in the oven at 60°C for 72 hr, weighed into separate portions (resource Rich = 1 g and resource Poor = 0.5 g), and placed in 5 mm mesh plastic bags. The leaves were then leached and conditioned for 2 weeks in running environmental water at 18°C. The nutritional quality of the leaves is known to increase during conditioning due to microbial colonization and assimilation of nutrients from the water (Boling et al., 1975; Marks, 2019). The average total microfungi biomasses on fully conditioned *Phragmites australis* leaves can reach up to 2%–5% of the leaf's weight (Van Ryckegem et al., 2006). For the behavioral experimental trials, the specimens were provided resources ad libitum (Li et al., 2007); however, they are selective foragers, known to feed on specific microfungi communities within the varied mix of fungi present in the leaves. Additionally, when their preferred microfungi are depleted or not available, they can consume other sources of microorganisms and leaves (Arsuffi & Suberkropp, 1989; Bärlocher & Kendrick, 1973). This approach more closely resembles their natural environment, as detritivores in transitional water ecosystems are often found in environments rich in dense detritus (Basset et al., 2001; Careddu et al., 2015).

### 2.4 Foraging behavior setup and measurements

**FIGURE 1:**
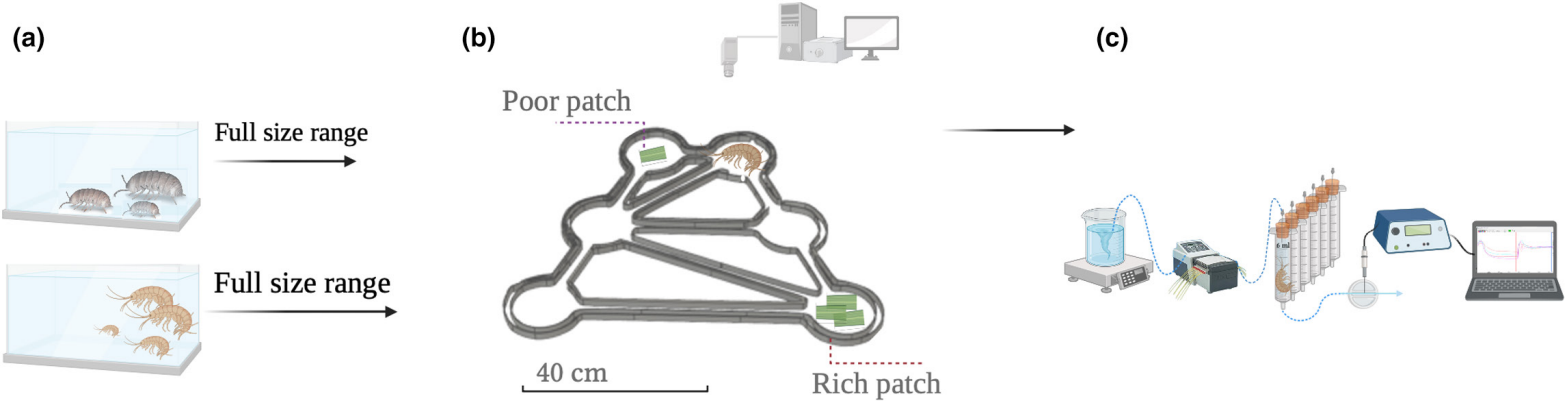
Schematic representation of the experimental design. (a) Maintenance of the model species. For each species, a full range of animal body masses was individually measured for behavior and metabolic rate. (b) The behavioral setup and experimental microcosm, consisting of one resource‐Rich patch, one resource‐Poor patch, and four empty patches. (c) The open‐flow respirometry setup for measuring individual metabolic rate. Figure created with BioRender.com, publication license HE26NPY18J.

The experimental system (ad hoc by Noldus Information Technology BV), consisting of three distinct microcosms, was set in an isolated and temperature‐controlled room (KW apparecchi scientifici, WR UR). Each microcosm, made of transparent Plexiglas, comprised six circular patches (13 cm in diameter, 3 cm high), connected by a network of channels (2.5 cm wide, 3 cm high) (Figure 1b). The microcosms were placed on top of a near‐infrared backlight source in order to have a high contrast which facilitated the specimen detection. Three infrared‐sensitive cameras (Basler, aca1300‐60gm) mounted above each microcosm to film the individual movement and its patch use (see Shokri et al. (2021) for the detailed experimental equipment). The combination of near‐infrared backlight and an infrared‐sensitive camera allowed us to conduct the experiments in the dark (complemented by a dim desktop light), effectively avoiding light reflections on the water surface that could interfere with image analysis.

Before behavior assessment, each of the specimen analyzed was kept unfed for 24 h in the controlled climatic room at 18°C. This was necessary to standardize specimens' resource requirements to a similar condition at the start of experimental trial (Shokri et al., 2021). For each experimental trial, 1 g dried weight of conditioned leaf fragments was placed in one patch, 0.5 g dried weight of conditioned leaf fragments was placed in another patch, thereby simulating a heterogenous resource distribution with two resource patches; called as “Rich” and “Poor” amount, and the other four patches were left “Empty” (Figure 1). Moreover, the distribution of the resource patches was randomized for each experimental trial to avoid any effect of microcosm geometry. The resource patches were placed in the microcosm 30 min before starting the experiment. Each experimental trial was performed on a single specimen foraging alone in the microcosm. The experimental trials were always conducted at the same time of the day (09:00 to 15:00) to prevent any possible effect of the circadian rhythms of the model organism. The recordings were started 10 min after the specimen was released into the microcosm, where it was free to move, and lasted 6 h. The video files were then processed by Ethovision XT 14 in batch acquisition mode, and the specimen was identified by the software as moving elements compared to the static background. A patch was considered to be “visited” once the specimen completely passes the whole channel, enters a neighboring patch, and persists in the patch for at least 30 s (see Shokri et al. (2021) for detailed method).

### 2.5 SMR and body mass measurements

After behavioral measurement, specimens were kept unfed individually for 24 h before the SMR (J day^−1^) measurements to standardize the conditions and to allow clearance of any food consumed during behavior trials. Twenty‐four hours is sufficient to complete digestion in the tested species (Welton et al., 1983) and to minimize the residual effects from the behavior experiment. Following the methods of (Glazier & Sparks, 1997; Wrona & Davies, 1984), the individual SMR was measured as oxygen consumption with specimens in a post‐absorptive resting state, at a constant temperature of 18°C. To assess their SMR, the animals were placed individually in Strathkelvin open‐flow system respirometers (Figure 1c). The respirometer measurement system includes a glass water tank (1 liter) filled with the same water characteristic as the acclimation aquaria, which was kept magnetically stirred and oxygen‐saturated throughout the experiment, using a digital ceramic magnetic stirrer (AREC.X). A peristaltic pump (Watson‐Marlow 205U, 12 channels) provided water flow to six respirometer chambers (6 mL volume), each containing a single individual. The experimenters ensured that the oxygen levels in the chambers never dipped below 80% saturation by maintaining a sufficient flow rate, also reducing the potential bias in the signal‐to‐noise ratio related to the chamber's volume. The flow rate of every chamber was measured by the chamber outflow volume of water per unit of time. An equilibration period of 3 h was fixed as the time required to reach a steady concentration of dissolved oxygen, which also enabled specimens to adapt to the respirometer chambers and reduce their spontaneous activity. Upon exit of the chambers, the water was pumped via silicone tubes to the Clark‐type microelectrodes (SI1302 Strathkelvin oxygen electrodes), where the oxygen concentration was continuously measured by an oximeter, with the data being recorded and stored using Strathkelvin software (SI, 929). The operators then read the dissolved oxygen partial pressure (∆torr) for each individual for 30 min: 15 min for the oxygen concentration curve (in the presence of a specimen: ppin) and 15 min for the blank (in the absence of specimens: ppout) (see Shokri et al. (2019) for the detailed method).

The oxygen consumed by each individual VO_2_ (μmol O_2_ h^−1^) was calculated as:

(1) $${\mathrm{VO}}_{2}=\left(\text{ppout}-\text{ppin}\right)\times {\mathrm{SO}}_{2}\times F,$$

where “ppout” is the partial pressure (torr) of dissolved oxygen in the outflow water of the blank (without specimens), “ppin” is the dissolved oxygen partial pressure (torr) of the respirometer chamber (with a specimen), *F* is the water flow rate (l h^−1^) and SO_2_ is the solubility coefficient of dissolved oxygen in water (μmol L^−1^ torr^−1^). For the assessment temperature, the solubility coefficient of dissolved oxygen (SO_2_) was obtained from a Loligo oxygen converter table. The rate of oxygen consumption was then converted to metabolic rate (J day^−1^) by using an oxyjoule equivalent of 0.45 J (μmol O_2_)^−1^ (Gnaiger, 1983), and by multiplying the resulting value by 24 h. After metabolic measurement, the animals were individually dried in an oven at 60°C for 72 h and then weighed on a microbalance (Sartorius MC5) to the nearest ±0.001 mg. Next, the ash weight was obtained by ashing the specimens at 450°C for 6 h. The obtained ash weight was then subtracted from the dry weight to calculate the individual ash‐free dry weight (M, mg AFDW). This allowed us to compare the body mass of the species by removing inorganic tissue since the amount of inorganic content varies among macroinvertebrate bodies.

### 2.6 Data analysis

The scaling of the individual SMR (J day^−1^) with individual body mass (M, AFDW mg) across species was assessed via linear regression. The response variable individual SMR and the explanatory variable body mass were log‐transformed in order to fit the size‐scaling relationship as a power law (Brown et al., 2004). We analyzed the behavioral patterns of the specimens in the experimental microcosm with reference to two descriptors of space and time use behavior: (1) cumulative space use and (2) average time spent in resource patches. Variation in the behavioral descriptors was analyzed across individual body mass (M), SMR, both within species and among individuals, irrespective of species identity (by pooling all individuals). To avoid multicollinearity between the explanatory variables SMR and M, two separate regression models were employed for each behavioral descriptor. The first model included M but not SMR as explanatory variables, and the other included SMR, excluding M.

The variation in cumulative space use behavior that is approximated as the total number of patches visited or revisited during the experiment, within species and among individuals (irrespective of species), was investigated by linear regression along the M gradients, and separately along the SMR gradient. Both the response variable of cumulative space use and the explanatory variable M were log‐transformed in order to fit the size‐scaling relationship as a power law (Jetz et al., 2004; McNab, 1963). For internal consistency and comparability, the explanatory variable SMR was also log‐transformed.

We quantified the average time spent as the average duration in minutes of visits to resource patches. The variation in average time spent was investigated by a linear regression along the M gradients, and separately along the SMR gradient within species and among individuals. Similarly to previous behavior descriptor, we log‐transformed the explanatory variable of M and SMR.

We further explored the relative importance of M and SMR, along with species characteristics and mobility, in relation to cumulative space use and average time spent. For each of these behavioral modes, linear regressions were conducted, considering M and species/mobility as predictors in one model, and SMR and species/mobility in another (see the Supporting Information). The uncertainty of model estimates was reported as the 95% Confidence Interval [lower‐upper]. All analyses were performed within the ‘R’ free software environment (R Core Team, 2019).

## 3 RESULTS

The specimens of *G. insensibilis* used in this experiment ranged from 5.24 to 17.47 mm in body length (on average 11.48 mm [± 4.14 SD]) and from 0.72 to 8.42 mg ash free dry weight in body mass (on average 4.34 mg [± 2.71 SD]). The specimen of *L. monodi* ranged from 3.15 to 10.58 mm in body length (on average 6.35 mm [± 1.76 SD]) and from 0.94 to 20.41 mg ash free dry weight in body mass (on average 5.88 mg [± 4.52 SD]). The average body mass (M, mg AFDW) was not significantly different between the two species (*t*‐test, *t* = 2.09, df = 47, *p* = .15).

### 3.1 Mass scaling SMR

**FIGURE 2:**
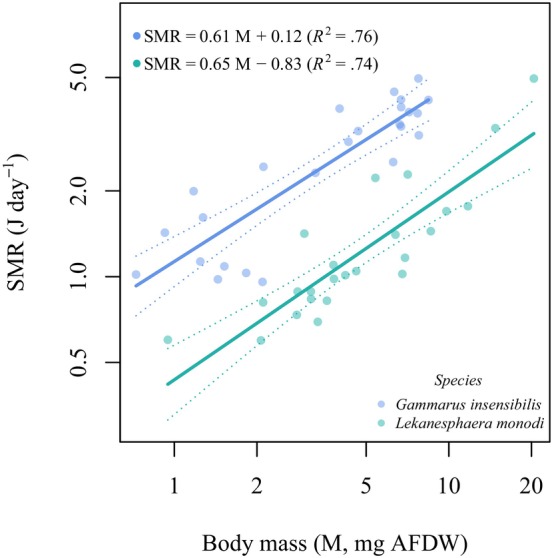
Standard metabolic rate (SMR, J day^−1^) in relation to body mass (M, mg AFDW) in two species, plotted on a log–log scale.

The metabolic rate allometrically scaled with body mass, similarly in both species with a scaling exponent of 0.61 [95% CI 0.47–0.76] in *G. insensibilis* and 0.65 [95% CI 0.49–0.83] in *L. monodi* (Figure 2). However, the scaling intercept of the relationship between metabolic rate and body mass was significantly higher in *G. insensibilis* compared to *L. monodi* (ANCOVA, *F* = 122.3, df = 46, *p* < .0001). This implies that individuals of *G. insensibilis* have a higher metabolic level per unit of mass than those of *L. monodi* (Figure 2).

### 3.2 Space use scaling with body mass and SMR

**FIGURE 3:**
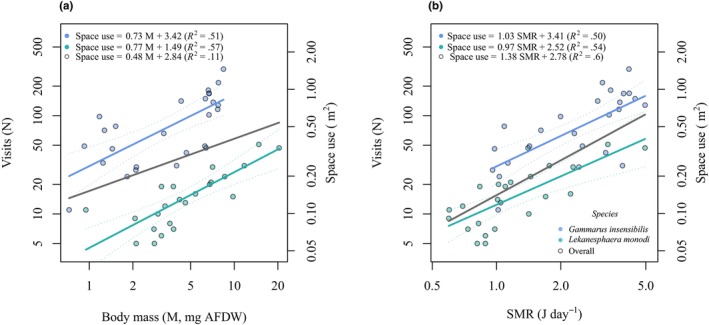
(a) Total number of visits to all patches in relation to body mass (M, mg AFDW) across species, plotted on log–log scale. The secondary *y*‐axis shows cumulative space use (m^2^), calculated as the overall surface area of patches that individuals visited. (b) Total number of visits to all patches in relation to standard metabolic rate (SMR, J day^−1^) across species, plotted on log–log scale. The secondary *y*‐axis shows cumulative space use (m^2^), calculated as the overall surface area of patches that individuals visited.

The cumulative space used was allometrically scaled with body mass similarly in both *G. insensibilis* (scaling exponent 0.73 [95% CI 0.42–1.04], 51.3% of explained variance) and *L. monodi* (scaling exponent 0.77 [95% CI 0.50–1.05], 57.3% of explained variance) (Figure 3). However, the scaling intercept of space use against body mass was significantly different between the two species, with *G. insensibilis* having a higher intercept (ANCOVA, *F* = 149.8, df = 46, *p* < .0001) (Figure 3). This indicates that individuals of *G. insensibilis* explored a larger space per unit of body mass compared to *L. monodi*.

The cumulative space used scaled isometric with SMR in *G. insensibilis* (scaling exponent 1.03 [95% CI 0.58–1.48], 49.8% of explained variance) and in *L. monodi* (scaling exponent 0.97 [95% CI 0.58–1.36], 53.9% of explained variance). Scaling exponents were not significantly different between the two species, while the scaling intercept of space use against SMR was significantly higher in *G. insensibilis* than in *L. monodi* (ANCOVA, *F* = 26.3, df = 46, *p* < .0001) (Figure 3).

Among individuals, regardless of species identity, body mass accounted for 10.8% of the variance in space use behavior (scaling exponent 0.48 [95% CI 0.07–0.88]; *F* = 6.20, df = 47, *p* = .021, AIC = 147.53). However, SMR, as a single descriptor among individuals, explained a substantial 59.9% of the observed variance in space use (scaling exponent 1.38 [95% CI 1.08–1.68]; *F* = 86.41, df = 47, *p* < .0001, AIC = 101.65), exceeding the amount of variance explained by body mass.

### 3.3 Giving up time scaling with body mass and SMR

**FIGURE 4:**
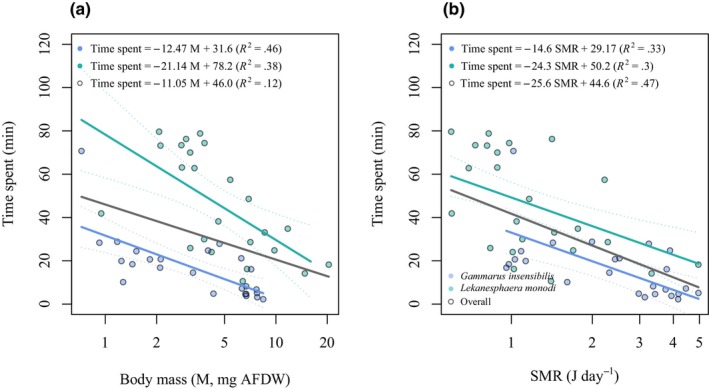
(a) The average time spent (min) in resource patches in relation to body mass (M, mg) across species. (b) The average time spent (min) in resource patches in relation to standard metabolic rate (SMR, J day^−1^) across species.

The average time spent in resource patches differed significantly between species, with *G. insensibilis* spending an average of 12.22 min visit^−1^ [± 22.86 SD] and *L. monodi* spending an average of 45.56 min visit^−1^ [± 23.67 SD] (*t*‐test, *t* = −5.24, df = 47 *p* < .001). The average time spent was negatively scaled with body mass, showing no significant difference in the scaling exponent between *G. insensibilis* (46.3% of the explained variance) and *L. monodi* (37.9% of the explained variance) (Figure 4). This shows that larger individuals tend to leave the resource patch earlier than smaller ones commonly in both species (Figure 4). However, the scaling intercept was significantly higher in *L. monodi* compared to *G. insensibilis* (ANCOVA, *F* = 58.2, df = 46, *p* < .0001), (Figure 4). This indicates that, per unit of body mass, *L. monodi* stayed in resource patches for a longer duration compared to *G. insensibilis*.

The average time spent showed a negative correlation with SMR with a similar scaling exponent in both species (32.9% explained variance in *G. insensibilis* and 29.6% explained variance in *L. monodi*) (Figure 4). This implies that individuals with a higher SMR tend to leave the resource patch earlier than individuals with a lower SMR. The scaling intercept was significantly higher for *L. monodi* compared to *G. insensibilis* (ANCOVA, *F* = 8.9, df = 46, *p* = .003) (Figure 4).

Among individuals, regardless of the species identity, while body mass accounted for 12.08% of the variance in the average time spent (*F* = 6.45, df = 47, *p* = .014, AIC = 450.28), SMR explained a greater portion of the variance 46.9% (*F* = 40.4, df = 47, *p* < .0001, AIC = 426.15).

## 4 DISCUSSION

Space and resource use behavior, which vary considerably among and within species, constitute a centerpiece to link individual function to higher‐order ecological processes. Overall, our empirical results showed that cumulative space use scaled positively with body mass and SMR, with the average time spent on resource patches falling as body mass and SMR increased. Our results further showed that within species, body mass and metabolic rate explained a similar amount of variation in behavior modes, while overall, pooling individuals regardless of species identity, SMR has stronger predictive power compared to body mass.

### 4.1 Mass scaling SMR

We found that the metabolic rate of both species was allometrically scaled with body mass. The allometric scaling of metabolic rate is in accordance with theoretical frameworks (Brown et al., 2004; Glazier, 2005) and empirical evidence (Killen et al., 2010; Shokri et al., 2022; Vignes et al., 2012).

Despite the similar scaling exponent of SMR against body mass in two species, our findings highlight the role of species identity and lifestyle in metabolic requirements, demonstrating that *G. insensibilis* individuals display a higher metabolic rate per unit of mass compared to those of *L. monodi*. This is likely because species of the *Gammarus* sp., which are generally fast‐moving or actively swimming, possess a higher metabolic rate to maintain their athletic performance, compared to the more sedentary or slow‐moving *L. monodi* species (Longo et al., 2016; Vignes et al., 2012). Our findings accord with previous studies that suggested factors beyond body mass, including morphology, ecology, and lifestyle, may influence variations in metabolic needs and energy expenditure (Glazier, 2005; Killen et al., 2010; White & Kearney, 2013).

### 4.2 Space use scaling with body mass and SMR

Individual cumulative space use was found to scale allometrically with the body mass of individuals in both species, implying that larger individuals explored more space than smaller ones. This finding is consistent with the theoretical framework (McNab, 1963), and empirical studies (e.g., Cozzoli et al., 2022; Minns, 1995; Udyawer et al., 2022). Correspondingly, we observed that in both species, individuals with high SMR cumulatively explored a greater portion of the space and resource. Specimens with higher SMR have more metabolically expensive organs and tissues, requiring more energy to maintain this level of metabolism (Auer et al., 2017; Metcalfe et al., 1995; Nilsson, 2002). This suggests that individuals with a higher metabolic rate are engaged in more extensive spatial exploration to access new resources. These efforts are presumably aimed at increasing food intake and fulfilling the energy requirements (see Biro et al., 2018). This finding is consistent with the performance energy‐management model and previous studies that showed a positive correlation between metabolic rate and space use (Biro & Stamps, 2010; Careau et al., 2011; Cozzoli et al., 2020; Metcalfe et al., 1995). However, this contradicts some other studies (e.g., Careau et al., 2008; Gifford et al., 2014), which sees a negative relationship between metabolic rate and behavior.

Within individuals of each species, our results suggest that body mass and SMR explained a similar proportion of variation in space use behavior. However, among individuals, irrespective of species identity, SMR demonstrated a stronger predictive power for space use behavior than body mass. In line with this, our further analyses, incorporating species identity alongside body mass and SMR, highlighted the substantial role of species identity and mobility characteristics when considering body mass as a predictor. Nevertheless, SMR, by encompassing to some extent the variation related to species identity and mobility, was shown to have greater predictive power in explaining behavioral patterns (see Supporting Information). This is likely because metabolic rate encompasses variation related to species‐specific, for example, ecology and lifestyle, thereby setting a continuum currency in predicting behavioral patterns among species (see also Brandl et al., 2022; Mathot et al., 2019). Our empirical finding in this regard accords with the meta‐analyses by Niemelä and Dingemanse (2018) and Mathot et al. (2019), as well as the review by Laskowski et al. (2022), which suggest that metabolic rate is more likely to be linked to aspects of behavior related to energy intake or expenditure than to other suggested state variables, such as body mass.

### 4.3 Giving up time scaling with body mass and SMR

The results of this study were derived from a patchy environment that comprised rich, poor, and resource‐absent patches. Although the total amount of resources in each experimental trial was overly abundant relative to the specimens' requirements, the tested species are known for their selective feeding behavior, prioritizing the consumption of a microfungus that is most palatable and provides the highest energy content (Basset & Rossi, 1990; Nelson, 2011). We found that individuals with larger body mass and higher SMR spent less time in resource patches in both species than individuals with smaller body mass and lower SMR. This might be because larger animals and those with higher metabolic rates have higher energy requirements and thus have a greater ingestion rate to meet their needs (Basset et al., 2012). Accordingly, compared to others, they likely depleted the most rewarding resources more quickly and sought a new patch offering resources with high energy returns, resulting in an earlier giving‐up time (Rosenfeld et al., 2015). Alternatively, larger animals, correspondingly with a higher SMR tend to leave a resource patch once the available resource reaches a level, known as the marginal value (Charnov, 1976), that can no longer fulfill their energy requirement, whereas smaller foragers and those with a lower SMR find it economically viable to continue exploiting the patch. This aligns with the findings of Spiegel et al. (2017), which demonstrated that in environments with discrete resource patches, specimens with higher metabolic rates (fast foragers, as per the pace of life syndrome (Réale et al., 2010)) disperse more readily and further, moving between resource patches more frequently and, as a result, having a larger home range. On the contrary, slower foragers, characterized by lower metabolic rates, engage in more methodical foraging, spending extended periods in a specific resource, and utilizing them down to lower levels of availability.

Although the overall average time spent similarly in both species was characterized by a negative SMR/mass dependency, it was observed that individuals of *L. monodi* tended to stay longer in a resource patch compared to *G. insensibilis* individuals who possess a higher SMR level. This observation supports the idea that metabolic rate, beyond body mass (since the species were similar in body mass), influences the behavioral strategies of resource use in these species. This could indicate that individuals of *L. monodi* species tend to forage the resource patch to a greater extent or have a lower resource harvest rate compared to *G. insensibilis*. The presence of distinct foraging strategies and differences in resource partitioning presumably can facilitate the coexistence of these species which live within the same environment (Chesson, 2000). The comparable amounts of variation in time spent in resource patches, within individual of each species as explained by body mass and SMR suggest that they are good descriptors of behavior at intraspecific level. However, among all the individuals, regardless of species, SMR showed to be a stronger predictor compared to body mass.

It must be noted that while experiments on the correlation between energy requirements and behavior, both interspecific and intraspecific, are clearly essential to test and develop relevant theoretical frameworks, limitations that might affect the generalization of the findings remain. Although the study was conducted on species within the same clade of crustaceans, further research involving a larger number of species, particularly those more closely related phylogenetically, would broaden the scope and substantiate the findings of our study. Additionally, the experiment was conducted within the species' thermal tolerance range; however, temperatures outside this range may alter the strength of the correlation between metabolic rate and behavior, highlighting the need for further investigation.

In summary, our empirical study provides insights into the factors influencing the space use and foraging behavior of animals in a patchy resource environment. We highlighted that metabolic rate might offer a generalized functional description of behavioral patterns that can encompass variations relating to body plan, species lifestyle, or identity. This suggests that understanding the dynamics and variations in individual metabolic rates, whether intrinsic or extrinsic, could shed light on predicting animal behavior related to energy intake or expenditure.

## AUTHOR CONTRIBUTIONS

**Milad Shokri:** Conceptualization (lead); formal analysis (lead); supervision (equal); writing – original draft (lead); writing – review and editing (lead). **Vanessa Marrocco:** Conceptualization (equal); data curation (equal); investigation (equal); methodology (equal); validation (equal); visualization (equal); writing – review and editing (equal). **Francesco Cozzoli:** Conceptualization (equal); investigation (equal); visualization (equal); writing – review and editing (equal). **Fabio Vignes:** Conceptualization (equal); methodology (supporting); validation (equal); visualization (equal); writing – review and editing (equal). **Alberto Basset:** Conceptualization (lead); funding acquisition (lead); investigation (equal); supervision (lead); visualization (equal); writing – review and editing (equal).

## CONFLICT OF INTEREST STATEMENT

The authors declare no competing or financial interests.

## Supporting information


Table S1.

## Data Availability

The data are accessible on the Open Science Framework, DOI:10.17605/OSF.IO/XZJQD.
