# Supplementary material for: The relative importance of metabolic rate and body size to space use behavior in aquatic invertebrates
Source: Ecol Evol. 2024 May 20;14(5):e11253. doi: 10.1002/ece3.11253 (PMC11103644; doi:10.1002/ece3.11253)
Supplement: Supplementary file 1 — Table S1. [file ECE3-14-e11253-s001.docx]

**Supplementary information**

Our results indicate that species identity and mobility, along with body mass, have a significant influence on individual behavioral patterns i.e. cumulative space use and average time spent. When body mass is used as a predictor, species identity and mobility explain a substantial portion of the variation in cumulative space use behaviour, accounting for 61% of it, though to a lesser extent (33% explained variation) when considering the SMR (Table S1). Importantly, SMR itself accounts for 44% of the explained variation, underscoring its greater predictive power compared to body mass, which accounts for only 17% (Table S1). Consistent with the findings for cumulative space use, SMR accounts for substantial variation in average time spent, beyond the contribution of body mass (Table S2). Furthermore, the relative importance of species/mobility was to a lesser extent when SMR served as the predictor (Table S2). This highlights that SMR, overall, to some extent, encompasses the variation associated with species identity and mobility, making it a more effective predictor of behavioral patterns.

Table S1. Summary of the linear regression of cumulative space used, approximated by the number of visits to all patches, as the response variable, with two sets of predictors: (a) body mass (M) and species/mobility, and (b) standard metabolic rate (SMR) and species/mobility. The relative importance of each fixed factor in explaining the variance of the dependent variable is reported as R^2^ part.

|  | (a) log (Visit) | | | |  | (b) log (Visit) | | | |  |
| --- | --- | --- | --- | --- | --- | --- | --- | --- | --- | --- |
| *Predictors* | *Estimates* | | *CI* | *p* | *R^2^ part* | *Estimates* | | *CI* | *p* | *R^2^ part* |
| (Intercept) | 3.40 | | 3.08 – 3.73 | **<0.001** |  | 3.44 | | 3.11 – 3.77 | **<0.001** |  |
| Slow mobility (*Lekanesphaera*) | -1.86 | | -2.17 – -1.55 | **<0.001** | 0.61 | -0.93 | | -1.30 – -0.57 | **<0.001** | 0.33 |
| log (M) | 0.74 | | 0.54 – 0.93 | **<0.001** | 0.17 |  | |  |  |  |
| log (SMR) |  | |  |  |  | 1.00 | | 0.71 – 1.29 | **<0.001** | 0.44 |
| Observations | | 49 | | |  | | 49 | | |  |
| R^2^ / R^2^ adjusted | | 0.79 / 0.78 | | |  | | 0.77 / 0.76 | | |  |

Table S2. Summary of linear regression between the average time spent in resource patches as the response variable, and two sets of predictors: (a) body mass (M) and species/mobility, and (b) standard metabolic rate (SMR) and species/mobility. The relative importance of each fixed factor in explaining the variance of the dependent variable is reported as R^2^ part.

|  | (a) Average time spent | | | |  | (b) Average time spent | | | |  |
| --- | --- | --- | --- | --- | --- | --- | --- | --- | --- | --- |
| *Predictors* | *Estimates* | | *CI* | *p* | *R^2^ part* | *Estimates* | | *CI* | *p* | *R^2^ part* |
| (Intercept) | 35.96 | | 26.39 – 45.53 | **<0.001** |  | 33.01 | | 22.81 – 43.22 | **<0.001** |  |
| Slow mobility (*Lekanesphaera*) | 34.52 | | 25.41 – 43.62 | **<0.001** | 0.43 | 16.21 | | 4.88 – 27.55 | **0.006** | **0.22** |
| log (M) | -16.06 | | -22.09 – -10.04 | **<0.001** | 0.18 |  | |  |  |  |
| log (SMR) |  | |  |  |  | -19.08 | | -28.01 – -10.15 | **<0.001** | **0.32** |
| Observations | | 49 | | |  | | 49 | | |  |
| R^2^ / R^2^ adjusted | | 0.60 / 0.59 | | |  | | 0.55 / 0.53 | | |  |
